# Myotubularin-related protein 14 suppresses cardiac hypertrophy by inhibiting Akt

**DOI:** 10.1038/s41419-020-2330-6

**Published:** 2020-02-20

**Authors:** Jie-Lei Zhang, Dian-Hong Zhang, Ya-Peng Li, Lei-Ming Wu, Cui Liang, Rui Yao, Zheng Wang, Sheng-dong Feng, Zhong-Min Wang, Yan-Zhou Zhang

**Affiliations:** 1 Department of Endocrinology, The First Affiliated Hospital of Zhengzhou University, Zhengzhou University, Zhengzhou, 450052 China; 2 Cardiovascular Hospital, The First Affiliated Hospital of Zhengzhou University, Zhengzhou University, Zhengzhou, 450052 China; 3 Department of Cardiology, The 7th People’s Hospital of Zhengzhou, Zhengzhou, China; 4 Department of Cardiology, FuWai Central China Cardiovascular Hospital, Zhengzhou, 450052 China

**Keywords:** Diseases, Metabolic syndrome

## Abstract

Cardiac hypertrophy (CH) is an independent risk factor for many cardiovascular diseases, and is one of the primary causes of morbidity and mortality in elderly people. Pathological CH involves excessive protein synthesis, increased cardiomyocyte size, and ultimately the development of heart failure. Myotubularin-related protein 14 (MTMR14) is a member of the myotubularin (MTM)-related protein family, which is involved in apoptosis, aging, inflammation, and autophagy. However, its exact function in CH is still unclear. Herein, we investigated the roles of MTMR14 in CH. We show that MTMR14 expression was increased in hypertrophic mouse hearts. Mice deficient in heart MTMR14 exhibited an aggravated aortic-banding (AB)-induced CH phenotype. In contrast, MTMR14 overexpression prevented pressure overload-induced hypertrophy. At the molecular level, prevention of CH in the absence of MTMR14 involved elevations in Akt pathway components, which are key elements that regulate apoptosis and cell proliferation. These results demonstrate that MTMR14 is a new molecular target for the treatment of CH.

## Introduction

Cardiac hypertrophy (CH) represents an adaptive response of the heart to an increased workload, and is also a well-established risk factor for cardiovascular mortality worldwide^1^. A multitude of factors, such as thyroid disorders, weakening of heart muscles, abnormal heart beat, protein deposition in the heart, mutations in sarcomeric proteins, and anemia, are known to contribute to CH^2^. Of note, CH is primarily characterized by excess protein synthesis, increased cardiomyocyte size, and thickened ventricular walls and is a major risk factor that promotes arrhythmia and heart failure^3^. Consistent with these characteristics, pathologic CH plays a compensatory role by increasing cardiac overload during the early stages. However, a sustained pathological hypertrophic response eventually leads to systolic dysfunction^4^. Therefore, CH is a predictor of many cardiovascular diseases and death in humans^5^. As a treatment option, inhibition of pathological CH has become a potential therapeutic target. Moreover, the signaling effectors underlying the pressure versus volume overload discrepancy remain to be clarified^6^.

Several mechanisms have been highlighted as potential contributors to the etiopathogenesis of CH, mostly based on common knowledge of pressure overload regulation. Signaling pathway protein kinases related to CH include AKT protein kinase B (PKB), PKA, Ca^2+^/calmoduli, and MAPKs, consisting of extracellular signal-regulated kinases (ERKs), c-Jun N-terminal kinases (JNKs), p38 MAPKs, and adenosine-activated protein kinase (AMPK), and nuclear factor-κB (NF-κB). However, although the aforementioned signaling pathways have been extensively characterized in pressure overload, improving our understanding of the mechanisms responsible for CH is important, and new molecular pathways involved in volume overload should be considered as potential therapeutic targets for cardiac hypertrophy.

Myotubularin-related protein 14 (MTMR14), a member of the myotubularin (MTM)-related protein family, is a novel phosphoinositide phosphatase, and inactivating MTMR14 mutations have been found in human centronuclear myopathy^7,8^. Previous studies have reported that MTMR14 is also involved in the regulation of the aging process, normal muscle performance, and autophagy^9^. Deletion of MTMR14 in mice disrupts calcium homeostasis and causes a muscle disorder^10^. Furthermore, MTMR14 deficiency caused late-onset inflammation and metabolic dysfunction^11^. Consistent with these findings, MTMR14 knockdown promotes apoptosis and inhibits migration in liver cancer cells^7^. Studies have also demonstrated that mice lacking MTMR14 show accelerated high-fat diet-induced lipid accumulation and inflammation^12^. At the molecular level, MTMR14 specifically dephosphorylates phosphatidylinositol 3,5-biphosphate (PtdIns(3,5)P2) and phosphatidylinositol 3-phosphate (PI3P), which are two of the seven phosphoinositides found in eukaryotic cell membranes^13^. The study demonstrated that MTMR14 expression is prominently increased in the hearts of mice that suffer from aortic-banding (AB)-induced CH, which suggests that MTMR14 may be involved in the regulation of CH. However, the specific role of MTMR14 in CH remains largely elusive.

Based on these observations, we performed a study, and we here present supporting evidence for MTMR14-mediated CH preservation. In this study, we found that MTMR14-deficient mice exhibit an aggravated AB-induced CH phenotype. Moreover, the prevention of CH in the absence of MTMR14 involves elevations in Akt signaling pathway components. These data provide evidence that MTMR14 is a potential therapeutic target for CH.

## Materials and methods

### Construction of animal models

All animal usage protocols were approved by Zhengzhou University. The related procedures were conducted in accordance with the National Institutes of Health Guide for the Care and Use of Laboratory Animals.

Cardiac-specific MTMR14-knockout (*MTMR14*-CKO) mice were produced using the Cre-loxP system. First, two single-guide RNAs (sgRNAs) targeting exon 3 of the MTMR14 gene were designed using an online CRISPR design tool (http://crispr.mit.edu/). The two sgRNAs were transcribed using the MEGAShortscript^™^ Kit (AM1354, Ambion). A Cas9 plasmid (pST1374-NLS-flag-linker-Cas9, Addgene, 44758) was transcribed using a T7 mMESSAGE mMACHINE Kit (AM1345, Ambion). The transcribed RNAs were purified with a miRNeasy Micro Kit (Qiagen, 217084) or a Cas9 RNeasy Mini Kit (Qiagen, 74104). Exon 3 of the MTMR14 gene was inserted into the backbone of the pBluescript SK(+) vector, and flanked by loxP sites and two homology arms to construct the donor vector. The Cas9 mRNA and sgRNAs, along with the donor vectors, were microinjected into zygotes using the FemtoJet 5247 microinjection system. The neonatal mice were subjected to genotyping to identify founder mice containing a floxed exon 3 on the same allele. The following primers were used to confirm that the two loxP sites were located on the same allele: forward: CCCCTAAGGAGAACCCTTGC and reverse: AGGCCCTGGTATTACATCCT. The founder mice were mated with C57BL/6J mice to obtain F1 offspring. Heterozygotes were genotyped by polymerase chain reaction (PCR) using the following primers: MTMR14-loxP-F: TGGCCTTTGCTAAATTTTCAGTG and MTMR14-loxP-R: GAATCAGAGCTGAAGCAGGC. Homozygous *MTMR14*-floxed mice were obtained by mating the heterozygotes. Then, the *MTMR14*-floxed mice were mated with tamoxifen-inducible TG mice [(Myh6-cre/Esr1*)1Jmk/J] that express MerCreMer driven by the cardiomyocyte-specific *α-MHC* promoter (α-MHC-MCM, The Jackson Laboratory, stock no. 005650) to obtain MTMR14^Flox/Flox^*-α-MHC-*MerCreMer mice. Cre-mediated recombination of the floxed alleles was induced in 6-week-old MTMR14^Flox/Flox^*-α-MHC-*MerCreMer mice through intraperitoneal injection of tamoxifen (25 mg/kg/day, Sigma, T-5648) for 5 consecutive days, leading to the generation of cardiac-specific MTMR14 conditional knockout mice. MTMR14-Flox mice serving as controls were given injections of an equal dose of tamoxifen.

To produce conditional MTMR14 transgenic (TG) mice, MTMR14 cDNA was inserted into the pCAG-loxP-CAT-loxP-lacZ vector to generate cardiac-specific MTMR14-TG mice. The vector was linearized and microinjected into zygotes to obtain the TG mice. Founder mice were identified through PCR with the primers pcag-seq-F (CATGTCTGGATCGATCCCCG) and MTMR14-seq-R (CGTGTCACCACTCCGAAGAA) and crossed with α-MHC-MCM TG mice to generate CAG-loxP-CAT-loxP-MTMR14/α-MHC-MCM mice. Then, conditional MTMR14-TG mice were obtained through intraperitoneal injection of 6-week-old CAG-loxP-CAT-loxP-MTMR14/α-MHC-MCM mice with tamoxifen for 5 consecutive days. MTMR14 expression was evaluated by Western blotting, and mice with successful cardiomyocyte-specific MTMR14 overexpression served as the MTMR14-TG mice. MTMR14-Flox mice treated with the same dose of tamoxifen acted as controls.

### Animal surgery

Cardiac hypertrophy was induced in mice through partial thoracic aortic banding, as previously described. Briefly, 8- to 10-week-old male mice were anesthetized with sodium pentobarbital via an intraperitoneal injection, and the left side of the chest was opened to expose the thoracic aorta through the second intercostal space after the toe pinch reflex disappeared. Subsequently, a specific needle (27-G for body weights (BWs) of 24–25 g or 26-G for BWs of 26–27 g) was placed on the thoracic aorta and ligated with 7-0 silk suture. Then, the needle was removed rapidly before closure of the thoracic cavity. Doppler analysis was conducted to evaluate the level of aortic constriction. Sham-operated animals underwent every step except for aortic ligation.

### Echocardiographic assessment

Mice were anesthetized with isoflurane (1.5–2%), and echocardiography was performed using a MyLab 30CV ultrasound system (Biosound Esaote Inc.) using a 15-MHz transducer. The left ventricular (LV) cavity size and LV wall thickness were acquired from at least three consecutive cardiac cycles. The end systole and end diastole were defined as the phases in which the smallest and largest LV area was obtained, respectively. The LV end-diastolic diameter (LVEDd), LV end-diastolic interventricular septum thickness (LVESd), fractional shortening (FS%), and ejection fraction (EF%) were measured from LV M-mode tracing with a sweep of 50 mm/s at the mid-papillary muscle level. FS was calculated using the formula FS% = (LVEDd–LVESd)/LVEDd × 100%.

### Histological analysis

Hearts were harvested 4 weeks after sham or AB surgery from experimental animals that had been perfused with a 10% potassium chloride solution to induce cardiac arrest at the end of diastole, and then fixed with a 10% formalin solution. After being embedded in paraffin, the hearts were cut into 5-μm transverse sections. The sections were stained with hematoxylin and eosin (H&E) to measure the myocyte cross-sectional area, and the abundance of collagen was assessed after Picrosirius red (PSR) staining. Fibrosis was measured as the average percentage of the positively stained area relative to the total area. More than 40 fields per group were examined. A quantitative digital image analysis system (Image-Pro Plus 6.0) was used for the image measurements.

### Cardiomyocyte culture and infection with recombinant adenoviral vectors

Neonatal rat cardiomyocytes (NRCMs) were isolated from the hearts of 1- to 2-day-old SD rats, as previously described^14^. The hearts were excised and digested with 0.03% trypsin and 0.04% type II collagenase. NRCMs were harvested and grown in DMEM/F12 (C11330, Gibco) supplemented with 20% fetal calf serum, 1% penicillin/streptomycin, and 5-bromodeoxyuridine (0.1 mM, to inhibit fibroblast proliferation) for 48 h, and then maintained under serum-free conditions for 12 h. Subsequently, the NRCMs were stimulated with angiotensin II (Ang II, 1 μmol/L) or the Akt inhibitor (AKTI) MK-2206 (Selleckchem, S1078, 1 μM) in phosphate-buffered saline (PBS) for an additional 24 or 48 h. To obtain MTMR14-overexpressing or -knockdown cells, the CDS region of the MTMR14 gene or MTMR14 shRNA, respectively, was cloned into a replication-defective adenoviral vector (pHBAD-U6-CMV-GFP) under the control of the cytomegalovirus (CMV) promoter to construct a recombinant adenovirus. Primary rat cardiomyocytes were infected with adenovirus, and GFP was used as a control. For infection, adenoviruses were used at a multiplicity of infection of 50 particles/cell for 24 h. The cells were used for the indicated analyses and experiments.

### Immunofluorescence staining

Immunofluorescence staining was performed to determine cell surface area. NRCMs were infected with the indicated adenovirus for 24 h, stimulated with PBS or Ang II (1 μmol/L) for 48 h under conditions of 37.0 °C and 5% CO_2_, and fixed with 4% formaldehyde. After permeabilization with 0.1% Triton X-100 in PBS and blocking with a 10% bovine serum albumin solution at room temperature, the cells were immunostained with an α-actinin antibody (05-384, Merck Millipore, 1:100 dilution), followed by staining with a fluorescent secondary antibody (donkey anti-mouse IgG [H + L] secondary antibody, A21202, Invitrogen, 1:200). Image-Pro Plus 6.0 software was used to measure cell surface area.

### IP assays

For IP assays, cultured HEK293T cells were co-transfected with the indicated plasmids for 24 h and lysed. Cell homogenates were incubated with constant agitation and then centrifuged. For each IP, 500 µl of the sample was incubated with 10 µl of Protein A/G-agarose beads (AA104307; Bestchrom, Shanghai, China) and 1 µg of the indicated antibody on a rocking platform (3 h at 4 °C). Finally, IPs were washed 5–6 times with cold IP buffer before adding 2× loading buffer. Cell lysates and IPs were eluted in loading buffer at 95 °C for 10 min, and western blotting analysis was carried out.

### Quantitative real-time (RT)-PCR and Western blotting

For the RT-PCR assay, total RNA was extracted from ventricular tissues or cells with TRIzol reagent (15596-026, Invitrogen). Then, cDNA was reverse transcribed from the RNA using a Transcriptor First Strand cDNA Synthesis Kit (04896866001, Roche). Quantitative real-time PCR was used to detect the expression of selected genes with SYBR Green PCR Master Mix (04887352001, Roche). Glyceraldehyde-3-phosphate dehydrogenase (GAPDH) was used as the reference gene. The primer pairs used in this study are listed in Supplementary Table 1.

For the Western blot analyses, total protein samples were extracted from ventricular tissues or cell samples using RIPA lysis buffer (50 mM Tris-HCl, pH 7.4, 150 mM NaCl, 1% Triton X-100 or NP-40, 1% sodium deoxycholate, 0.1% sodium dodecyl sulfate (SDS), and 1 mM EDTA) containing a protease inhibitor cocktail (4963124001, Roche), and the protein concentration was determined with a BCA Protein Assay Kit (23225, Pierce). After fractionation by using SDS polyacrylamide gel electrophoresis, the proteins were transferred to polyvinylidene fluoride membranes, which were blocked with 5% nonfat milk at room temperature for 1 h. After incubation with multiple antibodies overnight at 4 °C, the secondary antibodies were added the next day, and the bands were visualized using a Bio-Rad ChemiDoc XRS + system (Bio-Rad). The levels of the specific proteins were normalized to the levels of GAPDH. The antibodies used in this study are listed in Supplementary Table 2.

### Statistical analysis

The data are presented as the mean ± s.d. Comparisons between two groups were performed using two-tailed Student’s *t* tests. Differences among more than two groups were assessed using one-way analysis of variance (ANOVA) followed by the Bonferroni test (equal variances assumed) or Tamhane’s T2 test (equal variances not assumed). A value of *P* < 0.05 was considered to indicate a statistically significant difference. All statistical analyses were performed using SPSS (Statistical Package for the Social Sciences) software, version 21.0.

## Results

### MTMR14 expression is upregulated in hypertrophic mouse hearts

**Fig. 1:**
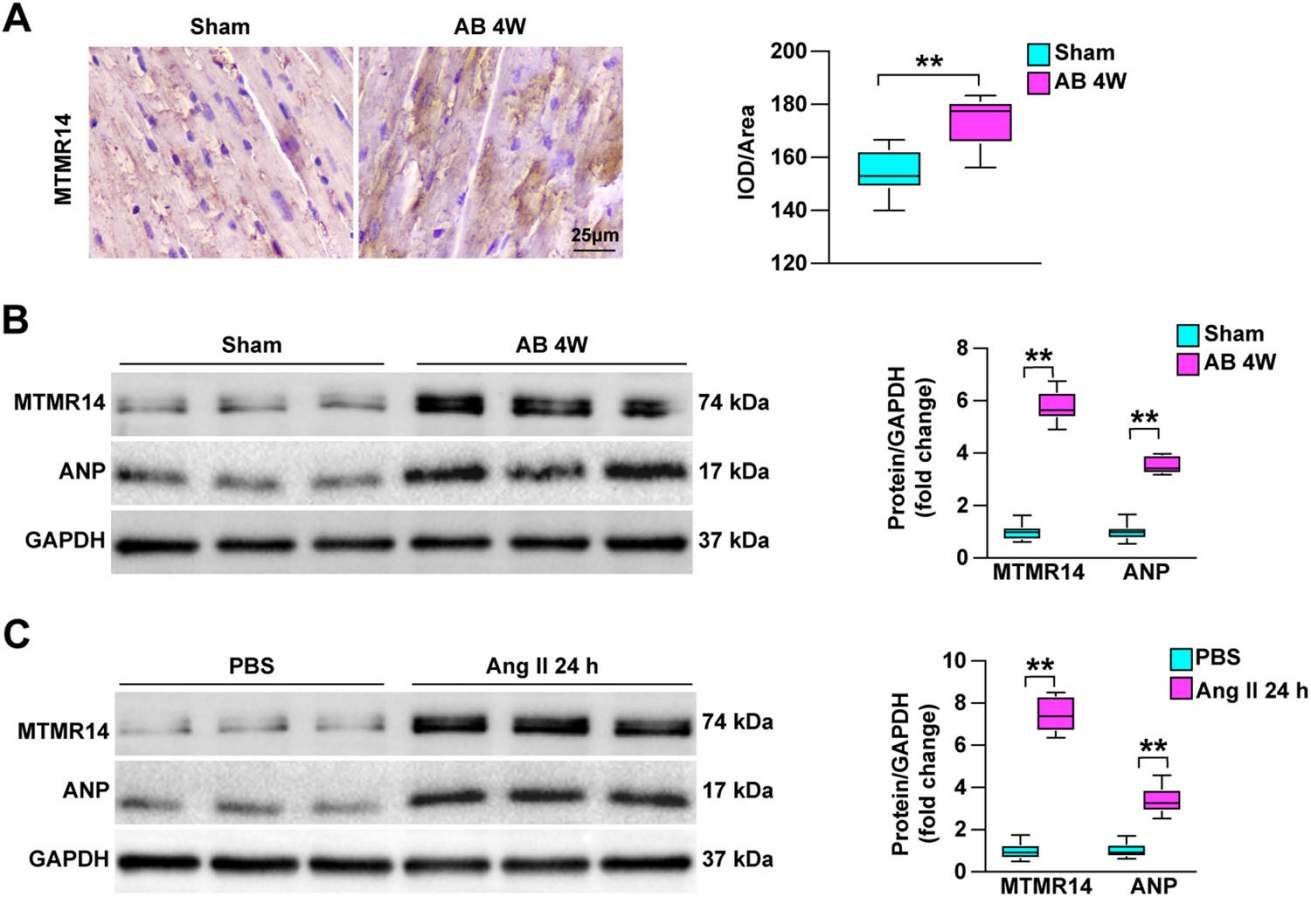
MTMR14 expression is upregulated by CH. **a** Representative images of immunohistochemical staining with an anti-MTMR14 antibody in heart slices from control mice and murine models of CH induced by aortic banding (AB) at the indicated time points; scale bar = 25 μm. **b** Western blot analysis of MTMR14 and ANP levels in the indicated groups. **c** Protein levels of MTMR14 and ANP in neonatal rat cardiomyocytes (NRCMs) treated with PBS or angiotensin II (Ang II, 1 μmol/L) for 24 h (*P < 0.05 vs. PBS). The protein levels were normalized to GAPDH for the Western blots in (**b**) and (**c**). The data are presented as the mean ± SD. *P < 0.05 and **P < 0.01; the data are from at least three independent experiments. For **b**, **c**, statistical analysis was carried out by one-way ANOVA.

To evaluate the link between MTMR14 and heart hypertrophy, we first tested the expression of MTMR14 in a mouse model of CH. As shown, MTMR14 protein expression was upregulated in the hearts of CH mice compared with those in control mice (Fig. 1a), as assessed by immunohistochemistry. MTMR14 protein levels were upregulated in the hearts of the hypertrophy models at 4 weeks after AB surgery compared with the hearts of the sham-operated mice. Simultaneously, as shown in Fig. 1b, the levels of the fetal gene ANP increased in the model mice, as assessed by Western blotting. In addition, the levels of both MTMR14 and ANP were upregulated in isolated NRCMs that were treated with angiotensin II (Ang II) for 24 compared with PBS-treated control NRCMs (Fig. 1c). Overall, the above data show that MTMR14 is involved in CH.

### Deficiency of MTMR14 aggravates CH

**Fig. 2:**
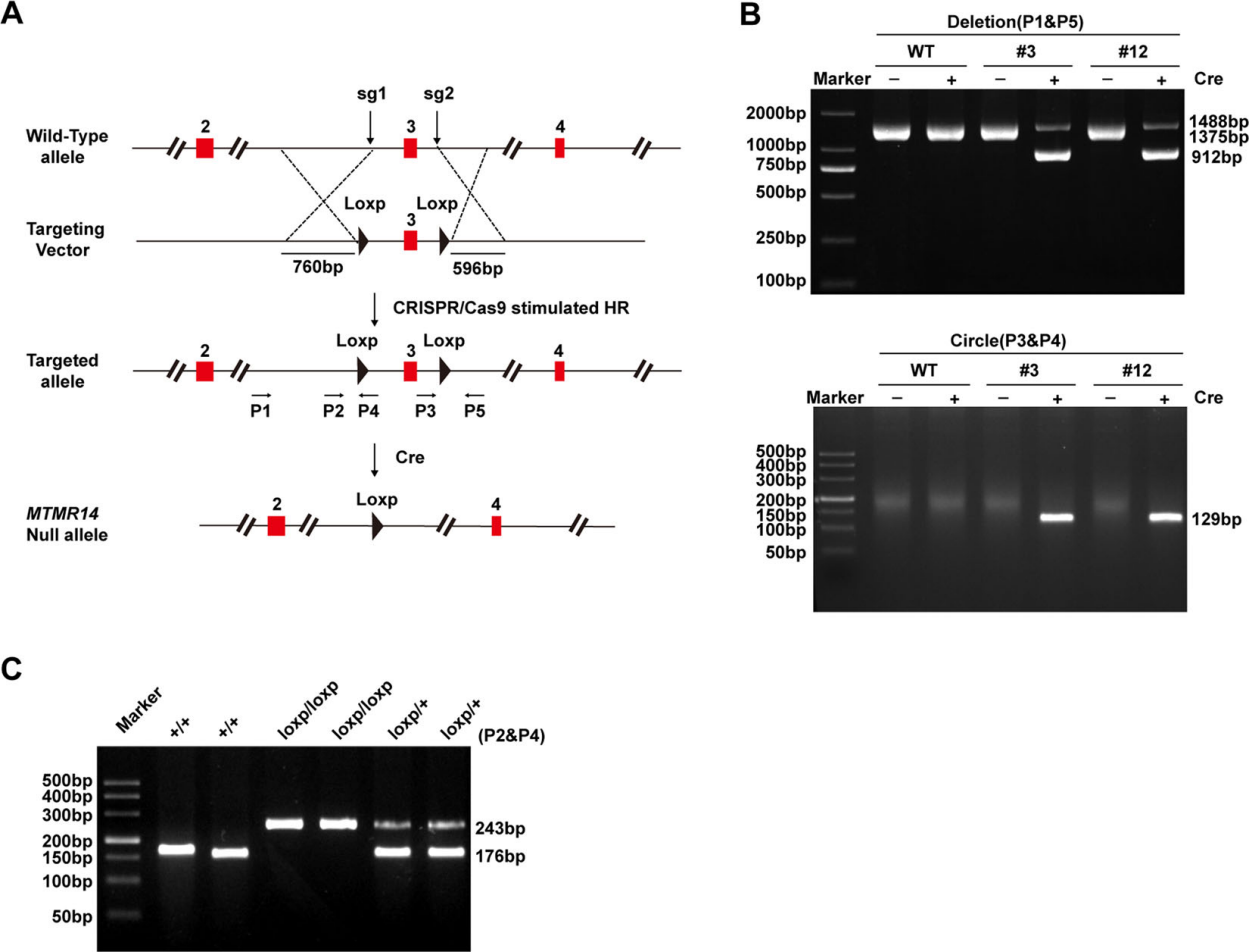
Construction of the cardiac-specific MTMR14-knockout (MTMR14-CKO) mouse strain. **a** Schematic workflow for the construction of the cardiac-specific MTMR14-knockout (MTMR14-CKO) mouse strain. **b** Mouse genotyping was confirmed by PCR. **c** Representative PCR amplification-mediated genotyping of MTMR14^loxp/loxp^, MTMR14^+/+^, and MTMR14^loxp/+^ mice.

**Fig. 3:**
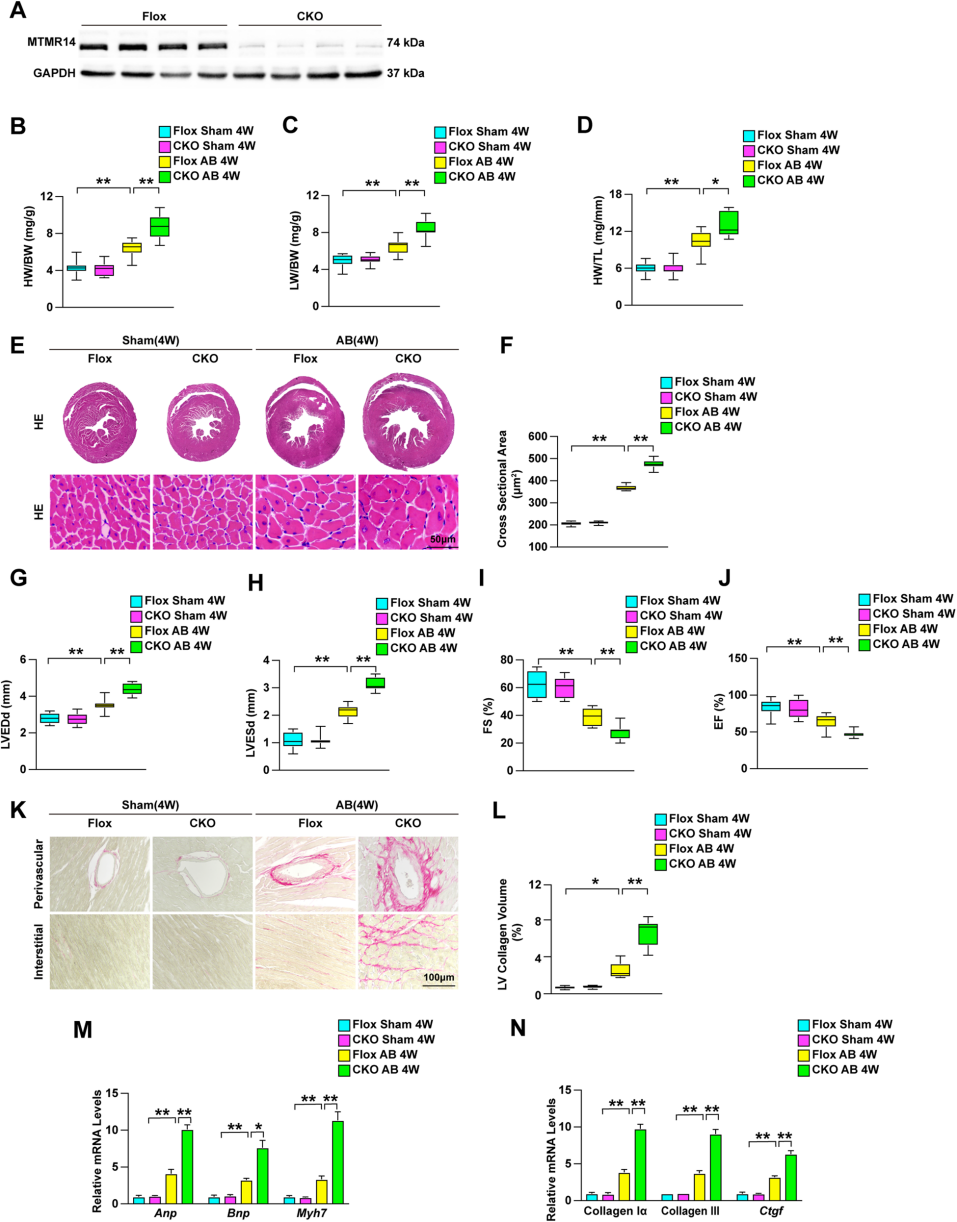
MTMR14 protects against pressure overload-induced CH. **a** Representative blot of MTMR14 in heart tissues from MTMR14-Flox and MTMR14-CKO mice (*n* = 6 in each group). **b**–**d** Cardiac parameters from the echocardiographic results, including HW/BW (**b**), LW/BW (**c**), and HW/TL (**d**) ratios in MTMR14-Flox and MTMR14-CKO mice 4 weeks after AB surgery (*n* = 10 in each group). **e**, **f** Histological analyses of heart sections stained with HE (*n* = 6 in each group; scale bar = 50 μm) (**e**) and results for cardiomyocyte area (*n* ≥ 100 cells in each group) (**f**). **g**–**j** LVEDd (**g**), LVESd (**h**), FS (**i**), and EF (**j**) in MTMR14-Flox and MTMR14-CKO mice 4 weeks after AB surgery (*n* = 10 in each group). **k**, **l** Representative PSR staining of the heart (*n* = 6 in each group; scale bar = 100 μm) (**k**) and statistical results for LV collagen volume (*n* ≥ 40 fields in each group) (**l**). **m**, **n** RT-qPCR analysis of hypertrophic markers (Anp, Bnp, and Myh7) (**m**) and fibrotic markers (collagen Iα, collagen III, and Ctgf) (**n**) in heart tissue samples (*n* = 4 in each group). The mRNA expression of the target genes was normalized to that of β-actin. The data are presented as the mean ± SD * *P* < 0.05 versus the MTMR14-Flox group and **P < 0.01 versus the MTMR14-Flox group.

In addition, to illuminate the effect of MTMR14 on CH in vivo, we established cardiac-specific MTMR14-knockout (MTMR14-CKO) mice. PCR and Western blot analyses were used to confirm the cardiac-specific deletion of MTMR14 (Figs. 2a–c and 3a). Next, MTMR14-Flox and MTMR14-CKO mice were subjected to AB surgery, and cardiac parameters were evaluated. As shown in Fig. 3b, the ratios of heart weight (HW) to BW were increased in MTMR14-CKO mice compared with MTMR14-Flox mice 4 weeks after AB surgery (Fig. 3b). In addition, the lung weight (LW)-to-BW and HW-to-tibial length (TL) ratios were increased significantly in the indicated groups compared with the control group (Fig. 3c, d). Moreover, HE staining of heart tissues showed that the hearts of MTMR14-CKO mice exhibited increased cross-sectional area compared with those of their MTMR14-Flox littermates (Fig. 3e, f). Furthermore, the MTMR14-CKO mice exhibited significantly increased LVEDd and LVESd values and decreased FS% values, as well as decreased EF values, compared with the MTMR14-Flox controls (Fig. 3g–j). Moreover, PSR staining of heart tissues showed that the hearts of MTMR14-CKO mice exhibited fibrosis in the perivascular and interstitial spaces compared with those of their MTMR14-Flox littermates (Fig. 3k–l). In parallel, the mRNA levels of CH- and fibrosis-related genes, such as Anp, Bnp, Myh7, collagen Iα, collagen III, and Ctgf, were significantly increased in the hearts of MTMR14-CKO mice compared with the hearts of the MTMR14-Flox controls (Fig. 3m, n). Taken together, these results show that MTMR14 deficiency contributes to pressure overload-induced CH.

### MTMR14 overexpression alleviates pressure overload-induced CH

**Fig. 4:**
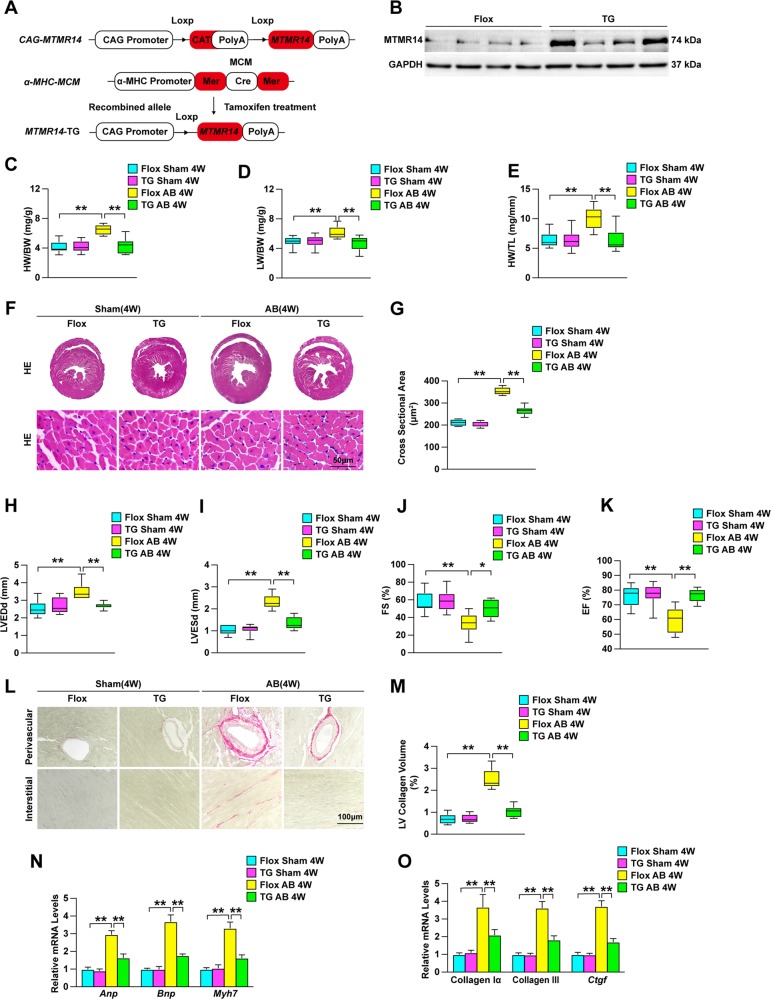
MTMR14 overexpression reduces pressure overload-induced CH. **a** Schematic workflow for the construction of the cardiac-specific MTMR14 transgenic (MTMR14-TG) mouse strain. **b** Representative blot of MTMR14 in heart samples from MTMR14-Flox and MTMR14-TG mice (*n* = 6 in each group). **c**–**e** Cardiac parameters from the echocardiographic results, including HW/BW (**c**), LW/BW (**d**), and HW/TL (**e**) ratios, in NTG and MTMR14-TG mice 4 weeks after AB surgery (*n* = 10 in each group). **f**, **g** Histological analyses of heart tissues stained with HE (*n* = 6 in each group; scale bar=50 μm) (**f**), and statistical results for cardiomyocyte cross-sectional area (*n* ≥ 100 cells in each group) (**g**). **h**–**k** LVEDd (**h**), LVESd (**i**), FS (**j**), and EF (**k**) in NTG and MTMR14-TG mice 4 weeks after AB surgery (*n* = 10 in each group). **l**, **m** Representative PSR staining of the heart (*n* = 6 in each group; scale bar = 100 μm) (**l**) and statistical results for LV collagen volume (*n* ≥ 40 fields in each group) (**m**). **n**, **o** RT-qPCR analysis of hypertrophic markers (Anp, Bnp, and Myh7) (**n**) and fibrotic markers (collagen Iα, collagen III, and Ctgf) (**o**) in heart tissue samples (*n* = 4 in each group). The data are presented as the mean ± SD. *P < 0.05 versus the NTG group and **P < 0.01 versus the NTG group.

To evaluate whether MTMR14 overexpression could reduce pressure overload-induced CH, we also established cardiac-specific MTMR14-TG (MTMR14-TG) mice (Fig. 4a, b). We evaluated cardiac parameters in NTG and MTMR14-TG mice that were subjected to AB surgery to further identify the regulatory role of MTMR14 in CH. As expected, the HW-to-BW ratios were decreased in MTMR14-TG mice compared with NTG mice 4 weeks after AB surgery (Fig. 4c). In addition, the LW-to-BW and HW-to-TL ratios were significantly decreased in the indicated groups compared with the control group (Fig. 4d, e). Furthermore, HE staining of heart tissues showed that the hearts of MTMR14-TG mice developed less cardiomyocyte hypertrophy than those of their NTG littermates (Fig. 4f, g). In addition, compared with the NTG controls, the MTMR14-TG mice exhibited prominently decreased LVEDd, LVESd, and FS values, as well as increased EF values (Fig. 4h–k). Furthermore, PSR staining of heart tissues showed that the hearts of MTMR14-TG mice developed less fibrosis than those of their NTG littermates (Fig. 4l, m). In parallel, the mRNA levels of Anp, Bnp, Myh7, collagen Iα, collagen III, and Ctgf were significantly decreased in the hearts of MTMR14-TG mice compared with the hearts of the NTG controls (Fig. 3n, o). Therefore, these results show that MTMR14 plays a beneficial role in pressure overload-induced CH.

### MTMR14 alleviates Ang II-induced cardiomyocyte hypertrophy in vitro

**Fig. 5:**
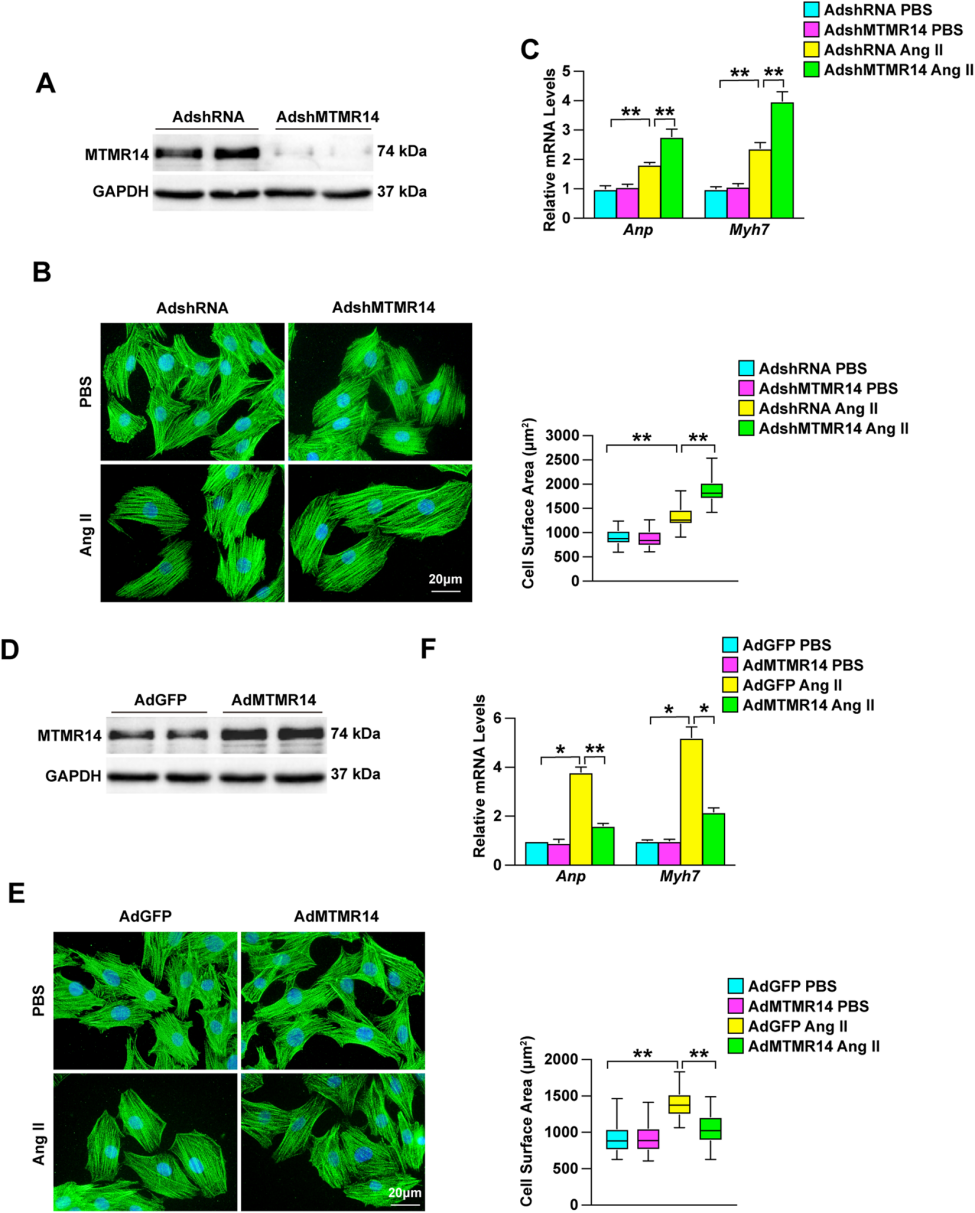
MTMR14 alleviates Ang II-induced cardiomyocyte hypertrophy in vitro. **a** Cellular lysates were analyzed by Western blotting to measure MTMR14 expression. **b** Representative images of cardiomyocytes infected with the indicated adenoviruses, and treated with PBS or Ang II for 48 h. Cardiomyocytes were identified by α-actinin staining (green), and nuclei were stained with DAPI (blue). Scale bars = 20 μm. **c** Quantitative results for the cell surface areas of cardiomyocytes infected with AdshMTMR14 or AdshRNA (control), and treated with PBS or Ang II for 48 h (*P < 0.05 vs. AdshRNA PBS, ^#^*P* < 0.05 vs. AdshRNA Ang II). **d** Cellular lysates were analyzed by Western blotting to measure MTMR14 expression. **e** Representative images of cardiomyocytes infected with the indicated adenoviruses and treated with PBS or Ang II for 48 h. Cardiomyocytes were identified by α-actinin staining (green), and nuclei were stained with DAPI (blue). Scale bars, 20 μm. Quantitative results for the cell surface areas of cardiomyocytes infected with AdMTMR14 or AdGFP (control), and treated with PBS or Ang II for 48 h, *P < 0.05 versus AdGFP PBS, ^#^*P* < 0.05 versus AdGFP Ang II. **f** Real-time PCR was used to determine the transcript levels of Anp and Myh7 in adenovirus-infected cardiomyocytes after treatment with PBS or Ang II for 48 h (*P < 0.05 vs. AdGFP PBS, ^#^*P* < 0.05 vs. AdGFP Ang II). The data are presented as the mean ± SD from at least three independent experiments. Statistical analysis was carried out by one-way ANOVA.

Since cardiomyocyte enlargement is the essential feature of cardiac remodeling, we further studied the particular role of MTMR14 in cardiomyocytes. MTMR14 knockdown mediated by an adenovirus was confirmed by western blot (Fig. 5a). Immunostaining with α-actinin showed that MTMR14 knockdown significantly exacerbated myocyte hypertrophy at 48 h after Ang II treatment (Fig. 5b). Consistently, RT-PCR results showed that the levels of Anp and Myh7 were dramatically upregulated in NRMCs infected with AdshMTMR14 compared with those infected with AdshRNA after Ang II treatment (Fig. 5c), showing that MTMR14 can directly alleviate the hypertrophic growth of isolated myocytes induced by Ang II. In contrast to MTMR14 knockdown, MTMR14 overexpression notably alleviated Ang II-induced cardiomyocyte enlargement, and significantly decreased the expression of fetal genes such as Anp and Myh7 (Fig. 5d–f). Collectively, these results demonstrate that MTMR14 alleviates Ang II-induced cardiomyocyte hypertrophy.

### MTMR14 inhibits AKT signaling in the pathogenesis of CH

**Fig. 6:**
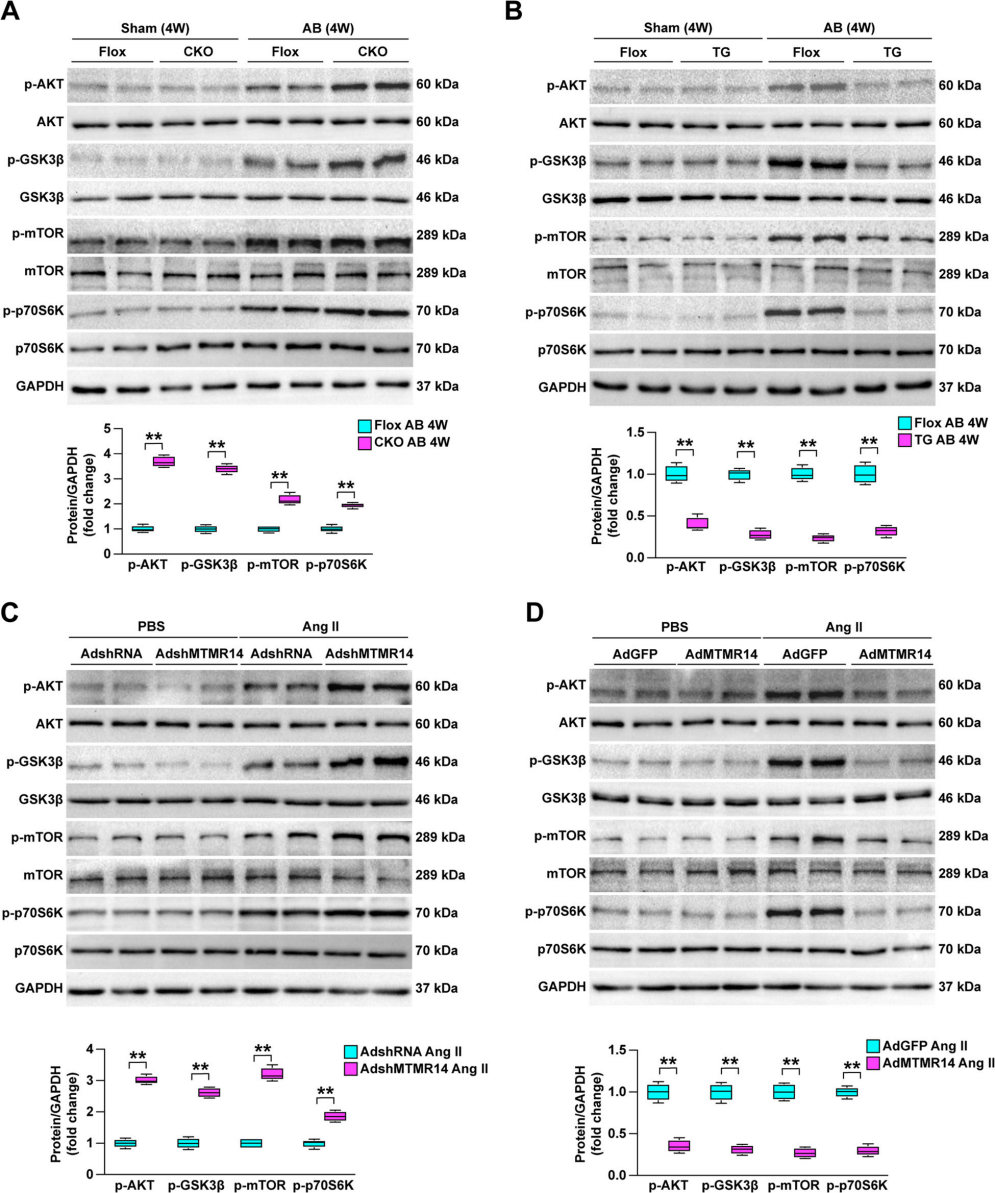
MTMR14 suppresses Akt/GSK3β/mTOR/p70-S6K signaling in the progress of CH. **a**, **b** The phosphorylated and total protein levels of Akt, GSK3β, mTOR, and p70-S6K in heart tissues from the indicated groups. For **a**, **b**, *P < 0.05 versus MTMR14-Flox sham or NTG sham, and ^#^*P* < 0.05 versus MTMR14-Flox AB or NTG AB. **c**, **d** The protein levels of phosphorylated and total Akt, GSK3β, mTOR, and p70-S6K in NRCMs infected with AdshRNA or AdshMTMR14 (**c**), or with AdGFP or AdMTMR14 (**d**), and treated with Ang II (*P < 0.05 vs. AdGFP PBS or AdshRNA PBS, ^#^*P* < 0.05 vs. AdGFP Ang II or AdshRNA Ang II). The data are presented as the mean ± SD from at least three independent experiments. Statistical analysis was carried out by one-way ANOVA.

Next, to further assess the molecular mechanisms underlying the effects of MTMR14, we tested the downstream pathways involved in MTMR14-mediated CH pathogenesis. Since the Akt signaling pathway has emerged as an essential intracellular pathway in CH, particularly AKT/GSK3β/mTOR pathways as regulators of CH^15–17^, we first studied the potential involvement of Akt signaling pathways in the pro-hypertrophic role of MTMR14. The Western blotting results showed that the phosphorylation levels of Akt, GSK3β, mTOR, and p70-S6K were significantly increased in hypertrophic hearts in MTMR14-CKO mice compared with the hearts of MTMR14-Flox controls subjected to AB surgery (Fig. 6a). In addition, the phosphorylation levels of Akt signaling pathway components were markedly decreased in response to AB surgery in TG mice compared with NTG controls (Fig. 6b). Similar results were also obtained in vitro. NRCMs were infected with AdshMTMR14 and AdMTMR14 and then subjected to Ang II intervention. As shown in Fig. 6c, Akt signaling pathway phosphorylation levels were increased when MTMR14 was knocked down (Fig. 6c), but were decreased when MTMR14 was overexpressed (Fig. 6d). Overall, these data indicate that MTMR14 suppresses activation of the Akt/GSK3β/mTOR/p70-S6K signaling pathways.

### MTMR14 regulates cardiomyocyte enlargement through its regulation of Akt

**Fig. 7:**
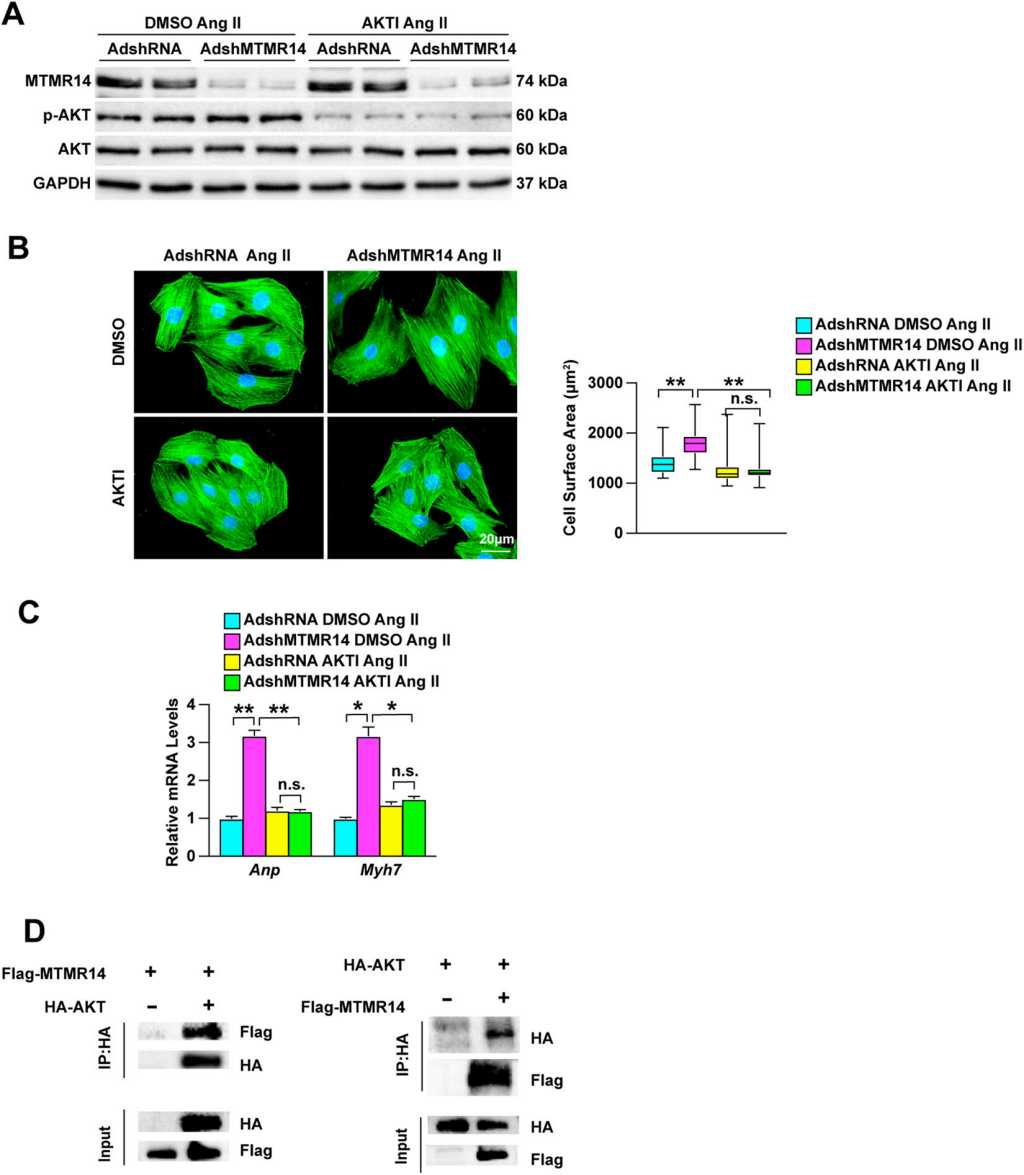
MTMR14 regulates cardiomyocyte enlargement through Akt. **a** The protein levels of MTMR14, p-AKT, and AKT were evaluated by Western blotting in primary cardiomyocytes that were infected with AdMTMR14 and treated with AktI following Ang II treatment. **b** Primary cardiomyocytes were infected with the indicated adenoviruses and treated with Ang II for 48 h. **c** mRNA levels of hypertrophic marker genes (Anp and Myh7) in primary cardiomyocytes transfected with the indicated adenoviruses and treated with Ang II for 48 h (*P < 0.05 vs. AdshRNA DMSO Ang II or AdshMTMR14 AKTI Ang II, **P < 0.01 vs. AdshRNA DMSO Ang II or AdshMTMR14 AKTI Ang II). **d** 293T cells were transiently transfected with Flag-MTMR14 and HA-AKT, and Western blotting was used to detect the interaction between MTMR14 and AKT. Data are presented as the mean ± S.D. from at least three independent experiments. Statistical analysis was carried out by one-way ANOVA.

Next, we evaluated whether Akt mediated the regulatory effect of MTMR14 on cardiomyocyte hypertrophy. We treated AdshRNA- or AdshMTMR14-infected primary cardiomyocytes with DMSO or AKT inhibitor MK-2206. Western blot results showed that MK-2206 completely blocked the activation of AKT induced by MTMR14 knockdown (Fig. 7a). In addition, immunofluorescence staining showed that cardiomyocyte hypertrophy potentiated by MTMR14 knockdown was completely blocked by MK-2206 (Fig. 7b). Furthermore, blocking Akt activity also suppressed the upregulation of ANP and Myh7 mRNA expression in MTMR14-knockdown cells (Fig. 7c). Next, we investigated how MTMR14 regulates the activation of AKT. We performed a series of IP experiments using the HEK293T cell line. IP experiments demonstrated that MTMR14 co-immunoprecipitated with AKT and vice versa (Fig. 7d). Taken together, these results demonstrate that a MTMR14-Akt pathway is directly involved in the regulation of myocyte hypertrophy, which plays a crucial role in CH regulation by MTMR14.

## Discussion

The heart undergoes adaptive changes in response to long-term overload, namely, myocardial hypertrophy. The correct expression of genes in cardiomyocytes is the basis for normal cardiac function. Thus, abnormal gene expression may cause heart dysfunction^5^. Here, interesting insights have been reported about the role of MTMR14 in CH. We support the notion that mice deficient in cardiac MTMR14 exhibit an exacerbated AB-induced CH phenotype. MTMR14 overexpression protected the mice in this study against pressure overload-induced CH. Mechanistically, protection against CH in the absence of MTMR14 involved activation of the Akt signaling pathway, and Akt was necessary for MTMR14 deficiency-related promotion of overload-induced CH. This study offers new insights into the function of MTMR14 as a biomarker or potential therapeutic target in the regulation of CH. Therefore, the use of strategies designed to target MTMR14 is of interest for the metabolic and contractile remodeling in cardiac diseases.

MTMR14 was first identified as a phosphatase specific to skeletal and cardiac muscle^12^. It has been reported to be a muscle-specific component, and lifelong MTMR14 deficiency in humans has been implicated in centronuclear myopathy^18^. The human protein MTMR14 shares high homology with the active myotubularin CX5R motif^19^. Recently, studies have shown that MTMR14 functions in health and disease, such as muscle fatigue, metabolism, and aging^20^. While an increasing number of studies have focused on the role of MTMR14 in regulating the maintenance of muscle signaling, the potential character of MTMR14 in cardiac muscle, especially in CH, remains largely unknown. Specifically, the predominant characteristics of CH are myocardial dysfunction and fibrosis, and increased intercellular muscle fibers^3^. Our study indicated for the first time that MTMR14 expression was obviously upregulated in CH, indicating that MTMR14 acted as a suppressor of the hypertrophic signaling network. Moreover, we demonstrated that the hearts of MTMR14-CKO mice exhibited significant cardiomyocyte hypertrophy and fibrosis in the perivascular and interstitial spaces. Further study on the correlations between MTMR14 and CH may provide guidance for the treatment of certain types of cardiac disease.

The pathogenesis of CH is driven by the responses of cardiomyocytes, and other resident cells of the myocardium, such as fibroblasts, endothelial cells, pericytes, and immune cells, as well as immune, inflammatory, and progenitor cells, were recruited from the circulation to different dynamic stimuli, including mechanical and nonmechanical stimuli, present in conditions of cardiac remodeling^3,21–23^. In the adult heart, instead of an increase in cardiomyocyte number, individual cardiomyocytes increase in size, and the heart develops hypertrophy to reduce ventricular wall stress and maintain function and efficiency in response to an increased workload^24^. At the cellular level, the typical characteristics of pathological CH are increased cardiac muscle cell size, segregation of sarcomere structures, enhanced protein synthesis, as well as fetal gene reexpression^25^. Moreover, cardiomyocyte metabolism and function are differentially regulated in CH. Impaired adaptation of energy metabolism during the hypertrophic response exacerbates pathological hypertrophy and increases cardiomyocyte death^24,26^. Accumulating studies have shown that dysfunction of mitochondrial biogenesis, maladaptive fuel shift, and reactive oxygen species are three primary contributors to cardiomyocyte abnormalities, ultimately resulting in CH^27^. The heart enlargement accompanying the above responses and inhibiting the concurrent signaling pathways could conceivably be important therapeutically. In our study, we demonstrated that cardiac-specific MTMR14 deficiency significantly increased the levels of fibrosis-related genes, such as Anp, Bnp, Myh7, collagen Iα, collagen III, and Ctgf, thus aggravating Ang II-induced cardiomyocyte enlargement both in vivo and in vitro. These observations provide the first evidence of how MTMR14 is beneficially involved in regulating the pathogenesis of CH. Whereas MTMR14 was knocked out in the liver, we found several inflammatory factors as TNF-α, NF-κB, as well as some cytokines were upregulated, which could decrease glucose expenditure in the muscles via other mechanisms. Our findings reveal a previously unknown function of MTMR14 associated with CH.

Akt is a serine/threonine kinase involved in the regulation of cardiac growth, proliferation, migration, and metabolism^28^. Akt signaling regulates the development of cardiac hypertrophy via the activation of hypertrophy-related genes^29^. Furthermore, alterations in Akt signaling play important roles in many cardiovascular pathological processes, such as atherosclerosis, cardiac hypertrophy, and vascular remodeling. Studies have shown that CH is associated with activation of the Akt/mTOR signaling pathway^30^. Moreover, PI3K promotes the activation of Akt, directly contributing to the process of cardiac remodeling^31^. In this study, we showed that the phosphorylation levels of Akt, GSK3β, mTOR, and p70-S6K were significantly increased in the hypertrophic hearts of MTMR14-CKO mice compared with the hearts of controls subjected to AB surgery. When Akt was inhibited, MTMR14 downregulation had no effect on cardiomyocyte hypertrophy following Ang II stimulation. Taken together, these findings show that MTMR14 regulates cardiomyocyte enlargement through Akt.

In summary, our data show that MTMR14 acts as a beneficial molecule in CH. In addition, our study contributes to a deeper understanding of the role of MTMR14 in cardiovascular diseases, and establishes a molecular link between MTMR14 and Akt in the regulation of myocardial remodeling and CH progression. Nonetheless, the impact of these molecular pathways remains mostly undefined in the context of cardiovascular pathology. Overall, the results suggest that ensuring the appropriate function of MTMR14 under different conditions may be efficient for the treatment of CH. Our study indicated for the first time that MTMR14 acted as a suppressor of the hypertrophic signaling network. MTMR14 overexpression alleviates pressure overload-induced CH, providing that MTMR14 is a potential therapeutic target for CH. Moreover, in our future study, MTMR14 acting as an exogenous gene can be introduced through AAV9 adeno-associated virus or adenovirus, which may have a therapeutic effect on CH as well as other cardiovascular diseases.

## Supplementary information

Supplementary Information
