# Supplementary material for: Myotubularin-related protein 14 suppresses cardiac hypertrophy by inhibiting Akt
Source: Cell Death Dis. 2020 Feb 20;11(2):140. doi: 10.1038/s41419-020-2330-6 (PMC7033093; doi:10.1038/s41419-020-2330-6)
Supplement: Supplementary file 1 — Supplementary Information [file 41419_2020_2330_MOESM1_ESM.docx]

**Supplementary Data**

**Myotubularin-related Protein 14 Suppresses Cardiac Hypertrophy by Inhibiting Akt**

Jie-Lei Zhang ^1*^, Dian-Hong Zhang ^2*^, Ya-Peng Li ^2*^, Lei-Ming Wu ^2^ , Cui Liang^2^, Rui Yao^2^, Zheng Wang^2^, Sheng-dong Feng^3^, Zhong-min Wang^4 †^, Yan-Zhou Zhang^2†^

Article Information:

^1^ Department of Endocrinology, the First Affiliated Hospital of Zhengzhou University, Zhengzhou University, Zhengzhou, 450052, China

^2^ Cardiovascular Hospital, the First Affiliated Hospital of Zhengzhou University, Zhengzhou University, Zhengzhou, 450052, China

^3^ Department of Cardiology, The 7th People’s Hospital of Zhengzhou, Zhengzhou, China.

^4^Department of Cardiology, FuWai Central China Cardiovascular Hospital, Zhengzhou 450052,

China

^*^ These authors contributed equally to this work.

**^†^ Correspondence authors**

**Yan-Zhou Zhang, M.D., Ph.D.**

Professor

Cardiovascular Hospital, the First Affiliated Hospital of Zhengzhou University, Zhengzhou University.

No.1 Jianshe East Road, Zhengzhou, Henan 450052, China;

Phone: 0371-67967662;

Fax: 0086-0371-67967661;

E-mail: zhangyanzhou2050@sina.com;

**Zhong-Min Wang, M.D., Ph.D.**

Professor

Department of Cardiology, FuWai Central China Cardiovascular Hospital, Zhengzhou, 450052, China

No.1 Fuwai Road, Zhengzhou, Henan 450052, China;

Phone:0371-58680123;

Fax: 0086-0371-58680123;

E-mail: [wangzhongmin001002@163.com](mailto:jz_dong@126.com);

**Key words:** Myotubularin-related protein 14; Cardiac Hypertrophy; Akt

**Financial support:** This work was supported by grants from National Natural Science Foundation of China (81770048; 81970242); Cooperative Project of Academy training Foundation of Zhengzhou University(2016-BSTDJJ-13).

**Conflict of interest:** None.

**Supplementary tables**

Supplementary table 1

| Antibody | Manufacturer | Catalog number | Source species | Dilution |
| --- | --- | --- | --- | --- |
| MTMR14 | AVIVA | OAAN02298 | rabbit | 1:1000 |
| ANP | PTG | 27426-1-AP | rabbit | 1:1000 |
| p-AKT | CST | 4060 | rabbit | 1:1000 |
| AKT | CST | 4691 | rabbit | 1:1000 |
| p-mTOR | CST | 2971 | rabbit | 1:1000 |
| mTOR | CST | 2983 | rabbit | 1:1000 |
| p-GSK3β | CST | 9322 | rabbit | 1:1000 |
| GSK3β | CST | 9315 | rabbit | 1:1000 |
| p-p70S6K | CST | 9208 | rabbit | 1:1000 |
| p70S6K | CST | 2708 | rabbit | 1:1000 |
| GAPDH | CST | 2118 | rabbit | 1:1000 |

Supplementary table 2

| Gene name | Forward primer (mouse) | Reverse primer (mouse) |
| --- | --- | --- |
| Anp | TCGGAGCCTACGAAGATCCA | TTCGGTACCGGAAGCTGTTG |
| Bnp | GAAGGACCAAGGCCTCACAA | TTCAGTGCGTTACAGCCCAA |
| β-Mhc | CAACCTGTCCAAGTTCCGCA | TACTCCTCATTCAGGCCCTTG |
| Collagen Iα | TGCTAACGTGGTTCGTGACCGT | ACATCTTGAGGTCGCGGCATGT |
| Collagen III | ACGTAAGCACTGGTGGACAG | CCGGCTGGAAAGAAGTCTGA |
| Ctgf | TGACCCCTGCGACCCACA | TACACCGACCCACCGAAGACACAG |
| Gapdh | ACTCCACTCACGGCAAATTC | TCTCCATGGTGGTGAAGACA |
|  |  |  |
| Gene name | Forward primer (rat) | Reverse primer (rat) |
| Anp | AAAGCAAACTGAGGGCTCTGCTCG | TTCGGTACCGGAAGCTGTTGCA |
| Myh7 | GTTTGCTGAAGGACACTCAAATCC | TTCTTCTTCTGGTTGATGAGGCTGG |
| Gapdh | TGTGAACGGATTTGGCCCTA | GATGGTGATGGGTTTCCCGT |

**Supplementary Figures**


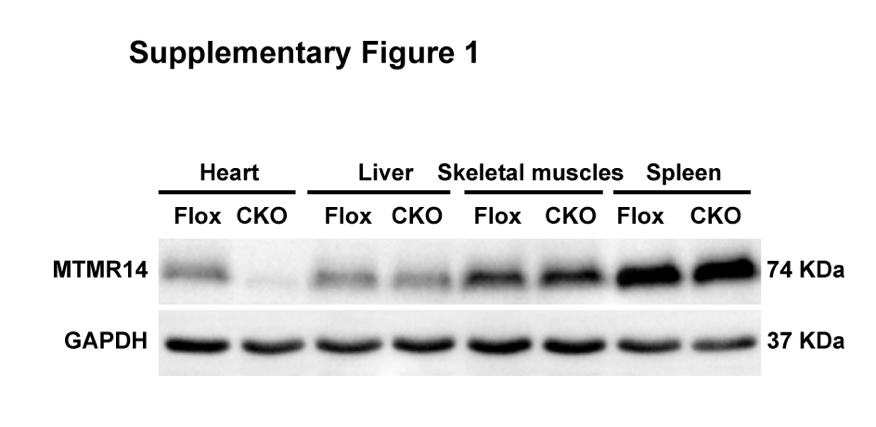


**Supplementary figure 1. MTMR14 expression in different tissues from MTMR14-Flox and MTMR14-CKO mice.** Representative blot of MTMR14 in different tissues from MTMR14-Flox and MTMR14-CKO mice (n=6 in each group).


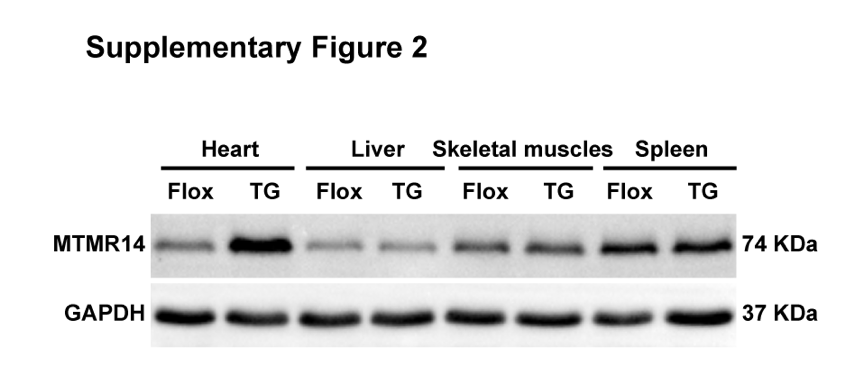


**Supplementary figure 2. MTMR14 expression in different tissues from MTMR14-Flox and MTMR14-TG mice.** Representative blot of MTMR14 in different samples from MTMR14-Flox and MTMR14-TG mice (n=6 in each group).
